# Suppression of lupus nephritis and skin lesions in MRL/*lpr* mice by administration of the topoisomerase I inhibitor irinotecan

**DOI:** 10.1186/s13075-016-1144-5

**Published:** 2016-10-22

**Authors:** Andreas Keil, Sean R. Hall, Meike Körner, Martin Herrmann, Ralph A. Schmid, Steffen Frese

**Affiliations:** 1 Department of Clinical Research and Division of General Thoracic Surgery, University Hospital Bern, Murtenstrasse 50, PO Box 44, , CH-3010 Bern, Switzerland; 2 Institute of Pathology, Länggasse, Bern, Switzerland; 3 Department of Internal Medicine 3 – Rheumatology and Immunology, Friedrich-Alexander-University Erlangen-Nürnberg (FAU), Erlangen, Germany

**Keywords:** Systemic lupus erythematosus (SLE), Lupus nephritis, Lupus-like skin lesions, Alternative treatment for SLE, Inhibitors of topoisomerase I, DNA relaxation, Anti-dsDNA binding

## Abstract

**Background:**

Since the precise mechanism for the pathogenesis of systemic lupus erythematosus (SLE) is unknown, no targeted therapies in addition to immunosuppression are available so far. We recently demonstrated that administration of the topoisomerase I (topo I) inhibitor irinotecan at extremely low concentrations reversed established lupus nephritis in NZB/NZW mice. While profound immunosuppression was absent, we proposed changes in DNA relaxation and anti-double-stranded (ds)DNA antibody binding as the underlying mechanism. To exclude that these effects were restricted to NZB/NZW mice, irinotecan was used in a genetically different strain of lupus-prone mice.

**Methods:**

MRL/*lpr* mice were treated with high- and low-dose irinotecan beginning at 8 weeks of age. Treatment was repeated every fourth week. In vitro, DNA was relaxed by recombinant topo I, and altered anti-dsDNA antibody binding was measured by enzyme-linked immunosorbent assay.

**Results:**

Administration of both high- and low-dose irinotecan prevented proteinuria and prolonged survival in MRL/*lpr* mice. Moreover, both concentrations of irinotecan significantly improved histopathology of the skin at 18 weeks of age. While only high-dose irinotecan diminished the numbers of plasmablasts and double-negative T cells, no changes in IgG-secreting cells or anti-dsDNA IgG were observed. In vitro, relaxation of DNA by topo I increased the binding of anti-dsDNA IgG but not the binding of anti-dsDNA IgM derived from the plasma of MRL/*lpr* mice.

**Conclusion:**

The beneficial effects of topo I inhibition in a second, genetically different strain of lupus-prone mice strongly implicate irinotecan as a new therapeutic option for human SLE.

## Background

Systemic lupus erythematosus (SLE) is a chronic autoimmune disease mainly affecting women of childbearing age. It is estimated that, in the USA, up to 275,000 adult women suffer from SLE [1]. The disease involves different organs, but immune complex glomerulonephritis most strikingly influences the course of SLE. Ten to thirty percent of patients with lupus nephritis progress to end-stage renal disease (ESRD) resulting in hemodialysis or kidney transplantation [2]. Due to the application of immunosuppressive drugs, the survival of patients with lupus-associated glomerulonephritis increased from a 5-year survival of 44 % in the 1950s to a 10-year survival of 88 % recently [3]. Despite these advances in the treatment of SLE, the life expectancy of patients with lupus and renal damage was recently demonstrated to be 23.7 years shorter compared to the general population [4]. Moreover, the incidence of ESRD associated with lupus nephritis has not decreased over the last years [5], indicating that current medication is insufficient to treat lupus nephritis.

Unselective immunosuppressive drugs remain the central strategy to control lupus nephritis. Medication consists of an induction therapy with cyclophosphamide and prednisolone or mycophenolate mofetil, followed by a maintenance therapy with azathioprine or mycophenolate mofetil [6, 7]. Major side effects of this medication are infections, and they bear the risk of malignancies whereupon cyclophosphamide also causes amenorrhea [8–10]. Furthermore, none of the newly developed biological agents such as rituximab, ocrelizumab, atacicept, or abatacept demonstrated beneficial effects in patients with active lupus nephritis [11–15]. Two other biologicals reached statistical significance for the improvement of moderate to severe forms of SLE. However, the benefit of belimumab and tabalumab were modest [16, 17], the “number needed to treat” was high, and other phase III studies hardly reproduced the first results [18, 19].

The precise mechanism for the pathogenesis of SLE is unknown. Therefore, no targeted therapies beside immunosuppression exist so far. However, a potentially targeted therapy for SLE was suggested by a new finding from our group which was generated by serendipity. We demonstrated that the topoisomerase I (topo I) inhibitor irinotecan efficiently suppressed murine lupus nephritis in New Zealand Black/New Zealand White (NZB/NZW) mice [20–22]. It was the first time that topo I was linked to the treatment of lupus nephritis.

Topo I is ubiquitously expressed and highly conserved [23, 24]. Its major function is the relaxation of supercoiled DNA in order to release torsional stress from DNA occurring during replication and transcription. To mediate DNA relaxation, topo I binds to DNA and cleaves one DNA strand, subsequently allowing the rotation of the cleaved strand around the other in a controlled reaction [25]. Afterwards, the nicked strand is re-ligated by topo I restoring intact double-stranded (ds)DNA in a relaxed state.

Inhibitors of topo I stabilize a normally very transient catalytic intermediate in which topo I is bound to one strand of the DNA, known as the topo I cleavable complex. When the cleavable complex is stalled by topo I inhibitors, re-ligation of the DNA is impossible [26]. The consequence of prevented re-ligation may vary; in proliferating cells, the stalled topo I cleavable complex can collide with replication forks leading to unrepairable DNA double strand breaks and apoptosis [27, 28]. For this reason, topo I inhibitors, like camptothecin and its synthetic derivative irinotecan, are widely used as anticancer drugs for several types of tumors [29]. In non-dividing cells, the treatment with topo I inhibitors results in the production of single-stranded (ss)DNA breaks reducing the replication capacity of a cell, although this is not lethal [26]. In addition to the induction of ssDNA and dsDNA breaks, topo I inhibitors were shown at least in vitro to inhibit DNA relaxation [30, 31].

In our first experiments, we applied concentrations of irinotecan that were similar to those used for chemotherapy in humans [20]. Although irinotecan reversed established lupus nephritis, there have been some concerns about using yet another chemotherapeutic for the treatment of SLE in predominantly young women. However, subsequent experiments by our group demonstrated that much lower dosages were still efficient to suppress SLE. We ended up with a dose more than 50 times lower than the dose used for chemotherapy in humans, which still enabled the successful treatment of established lupus nephritis in NZB/NZW mice [21]. These extremely low concentrations guided us to the hypothesis that inhibition of topo I might be a targeted therapy for SLE. While a profound immunosuppression was ruled out [20–22], we hypothesized in the beginning that induction of ssDNA breaks as a consequence of topo I inhibition is the underlying mechanism [32]. However, later we found that topo I alone increased binding of anti-dsDNA antibodies [21, 22] supposing that DNA relaxation is critically involved in the pathogenesis of SLE.

All previous experiments using irinotecan for the treatment of SLE were performed in NZB/NZW mice. To provide more evidence for first clinical trials treating human SLE we had to rule out that the demonstrated effects of irinotecan are restricted to NZB/NZW mice. We therefore introduced the MRL/*lpr* mouse model which is characterized by a fast and severe disease progression involving fatal glomerulonephritis, vasculitis, skin lesions, and massive lymphadenopathy [33, 34]. In these mice, we tested whether irinotecan has similar beneficial effects on lupus-like disease as shown before in NZB/NZW mice.

## Methods

### Mice

Female MRL/*lpr* and MRL/MpJ mice, aged 6 weeks, were purchased from The Jackson Laboratory and kept in isolated ventilated cages. Immediately after arrival, mice were randomly assigned to the respective groups (five animals per cage).

### Animal study: treatment of MRL/*lpr* with irinotecan

At 8 weeks of age, MRL/*lpr* mice were injected intraperitoneally with saline, or 1 or 25 mg/kg irinotecan (Campto®; Pfizer). MRL/MpJ mice treated with saline were used as controls. The volume of each injection was 10 ml/kg. Mice were treated three times per week. The treatment cycle was repeated after 4 weeks. Beginning at an age of 7 weeks, mice were monitored for proteinuria and body weight once a week. Proteinuria was measured with Albustix (Siemens Healthcare Diagnostics) and analyzed semiquantitively as grade 0 (negative), grade 1+ (≥30 mg/dl), grade 2+ (≥100 mg/dl), grade 3+ (≥300 mg/dl), and grade 4+ (≥2000 mg/dl) according to the manufacturer’s recommendations. The onset of proteinuria was defined as two instances of grade 4+ proteinuria occurring 1 week apart. Moreover, skin in the dorsal neck region, from the snout, and from the ears was scored individually in a semiquantitative manner using a score system from 0, for no lesion, to 2, for severe manifestation. Mice were killed when disease became severe (proteinuria grade 4+ and a body weight loss of ≥25 % from the onset of disease) and/or the total skin score was ≥4. The experiment was terminated when mice reached 37 weeks of age.

### Histopathology of kidney and skin sections

Kidney and skin obtained from the dorsal regions were fixed overnight in 4 % paraformaldehyde and embedded in paraffin. Standard protocols were used for hematoxylin and eosin, periodic acid-Schiff, and methenamine-silver staining.

For cryosections, tissue was immediately placed in OCT, snap frozen in liquid nitrogen and stored at –80 °C. Sections (6-μm thick) were fixed in acetone for 10 min before incubation with Alexa Fluor 488-conjugated goat anti-mouse IgG (H + L chain specific; Invitrogen). The kidney score of glomerulonephritis was assessed by an independent pathologist who was blinded to the groups using the International Society of Nephrology/Renal Pathology Society 2004 classification [35]. Skin was graded semiquantitatively according to Mizui et al. [36]; briefly, grade of acanthosis (none (0) to markedly thickened dermis (2)), hyperkeratosis (none (0) to strongly enhanced keratin (3)), fibrosis (normal (0) to markedly thickened dermal collagen (3)), inflammation (sparse (0) to substantial lymphocytic infiltrates (3)), and ulcer (absent (0) or present (1)).

### Isolation of splenocytes and lung cells

Spleens were harvested from mice and immediately transferred into ice-cold phosphate-buffered saline (PBS), and smashed on a sterile grid with a pestle. Cells were incubated in red blood cell lysis buffer for 2 min on ice, debris was allowed to settle out by centrifugation at 65 *g* for 2 min at 4 °C, and cells were re-suspended in RPMI 1640 supplemented with 10 % fetal calf serum (FCS). Cell viability was checked by trypan blue exclusion.

The left lobe of the lung was minced to small pieces and digested in RPMI 1640 without supplement containing 100 μg/ml Liberase (Roche) for 90 min at 37 °C and 5 % CO_2_. Digestion was stopped by adding fetal bovine serum (Gibco) to a final concentration of 10 %. Cells were passed through a 40-μm cell strainer, incubated in red blood cell lysis buffer for 2 min on ice and resuspended in RPMI 1640 media for cell counting and further analysis.

### Flow cytometry

Unspecific binding was blocked by incubating cells with Fc receptor-blocking monoclonal antibody (clone 2.4G2; BD Biosciences) for 10 min. Cells were then stained with the following antibodies specifically binding: CD3-PerCP/Cy5.5 (145-2C11; BioLegend), CD4-BV785 (RM4-5; BioLegend), CD8-BV421 (53-6.7; BioLegend), B220/CD45R-APC-Cy7 (RA3-6B2; BioLegend), CD138-BV605 (281-2; BioLegend), CD69-PE (H1.2 F3; BioLegend), and PD-1-PE-Cy7 (29 F.1A12; BioLegend). Dead cells were excluded by ZombieGreen™ (BioLegend) staining, and doublets by scatter analysis. Cells were measured on a LSRII flow cytometer (BD) and analyzed by FlowJo software. In most samples, a minimum of 1 × 10^5^ events were measured.

### Enzyme-linked immunosorbent spot (ELISpot) assay

Serial dilutions of splenocytes in RPMI 1640 supplemented with 10 % FCS were added to 96-well Multiscreen HTS Immobilon-P flat-bottomed plates (Millipore) pre-coated with goat anti-mouse IgG (Fc specific; Sigma-Aldrich) or goat anti-mouse IgM antibodies (BioLegend). After 4 h at 37 °C, plates were washed and incubated with alkaline phosphatase-conjugated anti-mouse IgG or alkaline phosphate-conjugated anti-mouse IgM (H + L chain specific, SouthernBiotech) for 1 h. Spots were developed with BCIP/nitroblue tetrazolium plus substrate (Sigma-Aldrich) and counted with an ELISpot reader (Autoimmun Diagnostika).

### Enzyme-linked immunosorbent assay (ELISA)

Ninety-six-well Nunc MaxiSorp plates were coated with 5 μg/ml goat anti-mouse IgG (Sigma) or anti-mouse IgM (SouthernBiotech) overnight at 4 °C. Plates were blocked with 1 % bovine serum albumin (BSA) in PBS for 1 h at 37 °C followed by incubation with diluted plasma samples for 1 h at 37 °C. Subsequently, plates were incubated with alkaline phosphatase-conjugated goat anti-mouse IgG or anti-mouse IgM (SouthernBiotech) for 1 h at 37 °C and developed with p-nitrophenyl phosphate (Sigma-Aldrich). Optical density was measured at 405 nm with a reference filter at 490 nm. Sample concentrations were calculated using a standard curve of purified mouse IgG (Sigma) and IgM (SouthernBiotech).

To determine anti-dsDNA antibodies, calf thymus DNA (Invitrogen) was passed through a Millex-HA 0.45-mm filter (Millipore) to remove any ssDNA fragments. Maxisorp plates were half-coated with 100 mg/ml calf thymus DNA in PBS overnight at 4 °C. Plates were blocked with PBS containing 1 % BSA for 1 h at 37 °C. Diluted plasma samples were incubated at 37 °C for 1 h. Bound anti-dsDNA autoantibodies were detected as described above for total IgG or IgM ELISA, respectively.

### DNA modification

Calf thymus DNA (50 mg/ml) was incubated with 4 μg/ml recombinant human topo I (Creative Biomart) in 50 mM Tris HCl (pH 7.5), 50 mM KCl, 2 mM dithiothreitol (DTT), and 1 mM EDTA for 30 min at 37 °C. Then, 30 ml of sample per well was used for coating a 384-well Nunc Maxi-Sorp plate overnight at 4 °C. Plates were blocked with PBS containing 1 % casein (Pierce) for 1 h at 37 °C. Next, depending on the experiment, diluted plasma samples from MRL/*lpr* mice were added to the plates for 1 h at 37 °C. Bound antibodies were detected as described above for anti-dsDNA IgG and IgM ELISA.

### Statistical analysis

Data were analyzed using either one-way or two-way analysis of variance (ANOVA) followed by Bonferroni’s post-hoc test, respectively. Some data were assessed by paired *t* test. Survival data were analyzed using the Mantel-Cox log rank test. For all tests, the software GraphPad Prism version 6.0 was used. *P* values <0.05 were considered as significant.

## Results

### Irinotecan attenuated symptoms of lupus-like disease and prolonged survival in MRL/*lpr* mice

MRL/*lpr* mice possess a rapid and severe form of lupus-like disease. For clinical monitoring, the involvement of the kidneys with proteinuria and the involvement of skin with alopecia, pruritus, and necrotic lesions are important. Both organ contributions influence the survival of these mice, in contrast to the NZB/NZW model preferably showing renal manifestations.

**Fig. 1:**
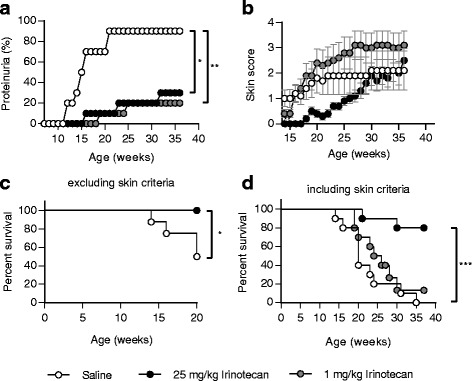
Suppression of SLE by irinotecan in MRL/*lpr* mice. Beginning at 8 weeks of age, MRL/*lpr* mice (*n* = 10 per group) were treated with saline, or 25 mg/kg or 1 mg/kg irinotecan. Treatment was performed three times a week and repeated every fourth week. **a** Frequency of proteinuria measured as grade 4+ at two consecutive weeks. **b** Skin score semiquantitatively assessed at the snout, ears, and dorsal region. Percent survival, showing animals sacrificed **c** due to lupus nephritis and excluding mice sacrificed due to skin lesions (*n* = 8 for saline, *n* = 10 for 25 mg/kg irinotecan, and *n* = 7 for 1 mg/kg irinotecan) or **d** including both lupus nephritis and skin lesions as sacrifice criteria (*n* = 10 for each group). Values represent the mean ± SEM. *P < 0.05, **P < 0.01, versus saline-treated MRL/*lpr* mice by two-way analysis of variance corrected by Bonferroni’s multiple comparisons test (**a** and **b**) or Mantel-Cox log rank test (**c** and **d**)

Mice were treated with high- and low-dose irinotecan from 8 weeks of age. The dosages for high- and low-dose irinotecan were obtained from previous experiments in NZB/NZW mice. As described before, low-dose irinotecan corresponds to a dose which is 50 times lower than the dose used for chemotherapy in humans [20–22]. Saline-treated MRL/*lpr* mice started to develop severe proteinuria from 12 weeks of age. At 21 weeks of age, grade 4+ proteinuria measured at two consecutive weeks affected 90 % of saline-treated animals. In contrast, both high- and low-dose irinotecan significantly prevented the onset of proteinuria from 16 weeks of age (Fig. 1a). Looking at the clinically determined skin score, only high-dose but not low-dose irinotecan suppressed the onset of detrimental skin lesions (Fig. 1b). Improvement of the skin score mediated by high-dose irinotecan was statistically significant until 27 weeks of age. Notably, the graph in Fig. 1b implies that MRL/*lpr* mice treated with low-dose irinotecan developed more severe lupus-like skin lesions than saline-treated animals. However, since saline-treated MRL/*lpr* mice showed a rapidly progressing glomerulonephritis in contrast to irinotecan-treated animals, they had to be sacrificed before they could reach higher levels of skin lesions.

Excluding animals that were sacrificed due to the skin score, both high- and low-dose irinotecan demonstrated a significantly improved survival at 20 weeks of age (Fig. 1c). When including all animals, only MRL/*lpr* mice treated with high-dose irinotecan demonstrated an increased survival of 80 % compared to 0 % of saline-treated mice and 10 % of animals treated with low-dose irinotecan at 37 weeks of age (Fig. 1d).

**Fig. 2:**
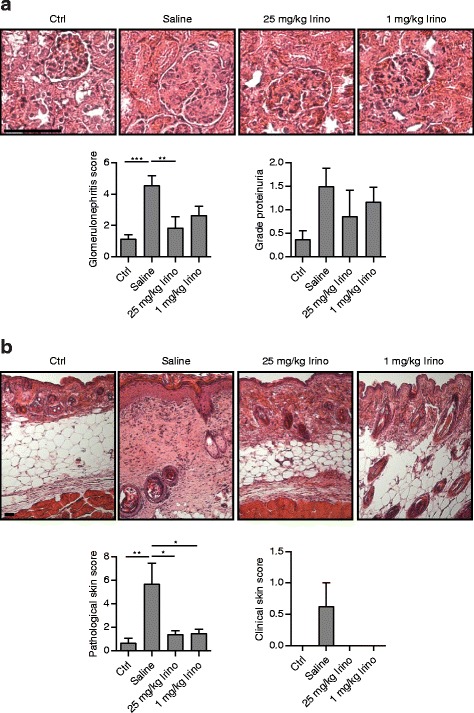
Improvement of kidney and skin score in MRL/*lpr* mice treated with irinotecan (*Irino*). MRL/*lpr* mice receiving saline (*n* = 7), 25 mg/kg irinotecan (*n* = 7), or 1 mg/kg irinotecan (*n* = 6) were sacrificed after the third treatment cycle (at an age of 18 weeks). MRL/MpJ mice treated with saline (*n* = 7) were used as controls (*Ctrl*). **a** Kidney sections and **b** skin sections from the dorsal region were scored by an independent pathologist in a blinded manner. Bars show the mean ± SEM. *P < 0.05, **P < 0.01, ***P < 0.001, versus saline-treated MRL/*lpr* mice by one-way analysis of variance corrected by Bonferroni’s post hoc test. Clinically determined proteinuria (**a**) and skin score (**b**) of these animals are shown from 17 weeks of age

For functional measurements, other groups of MRL/*lpr* mice were sacrificed at 18 weeks of age. MRL/MpJ mice were used as controls. Determination of the kidney score revealed an amelioration of glomerulonephritis for both irinotecan concentrations; however, only high-dose irinotecan showed a significant improvement (Fig. 2a). The skin score at 18 weeks of age assessed on paraffin sections by a blinded pathologist demonstrated a suppression of lupus-like skin lesions for both irinotecan-treated groups (Fig. 2b). Remarkably, when looking at the clinically determined parameters in Figs. 1 and 2, we noticed some differences between the groups of mice at 17 weeks of age: living saline-treated animals in Fig. 1 showed a proteinuria of 2.6 (mean, data not shown) while saline-treated mice in Fig. 2 presented a proteinuria of 1.5 (mean) at the same age.

### Immunomodulatory effects of irinotecan treatment in MRL/*lpr* mice

**Fig. 3:**
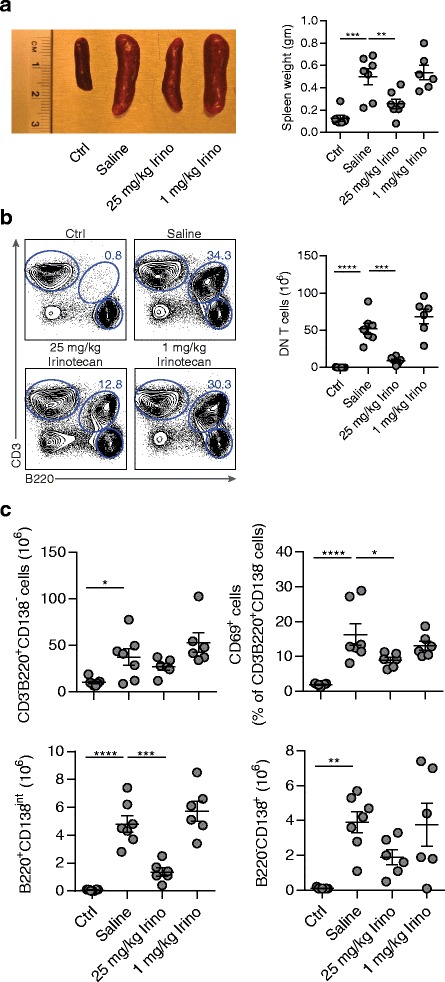
Prevention of splenomegaly by high-dose irinotecan (*Irino*). Spleens from 18 week-old MRL/*lpr* mice treated with saline (*n* = 7), 25 mg/kg irinotecan (*n* = 6), or 1 mg/kg irinotecan (*n* = 6) were analyzed by flow cytometry. MRL/MpJ mice treated with saline (*n* = 7) were used as controls (*Ctrl*). **a** Representative image of the spleen from each treatment group (*left*) and the spleen weights (*right*). **b** Representative bivariate contour plots from each treatment group showing the subpopulation of T cells double negative for CD4 and CD8 and positive for CD3 and B220 (*DN T cells*). Cells were gated for live, singlets, and negative for CD4 and CD8 expression. (*Right*) Total number of splenic DN T cells. **c** (*Top left*) Numbers of CD3^–^B220^+^CD138^–^ B cells and (*top right*) the expression of CD69. (*Bottom left*) Numbers of CD3^–^B220^+^CD138^+^ plasmablasts and (*bottom right*) CD3^–^B220^–^CD138^+^ plasma cells. Each symbol represents an individual mouse; bars show mean ± SEM.*P < 0.05, **P < 0.01, ***P < 0.001, ****P < 0.0001, versus saline-treated MRL/*lpr* mice by one-way analysis of variance with Bonferroni post hoc test

MRL/*lpr* mice bear a mutation in the Fas receptor (CD95). As a consequence, autoreactive immune cells fail to undergo apoptosis and accumulate in lymphoid structures [37]. We therefore examined how treatment with irinotecan influenced the immune system of MRL/*lpr* mice. Analysis of the spleen from mice sacrificed at 18 weeks of age described above revealed clear signs of splenomegaly in saline-treated MRL/*lpr* mice compared to MRL/MpJ mice. This effect was significantly reduced in MRL/*lpr* mice treated with high-dose irinotecan, whereas low-dose irinotecan had no influence on splenomegaly (Fig. 3a). In addition to splenomegaly, a massive proliferation of CD4/CD8 double-negative (DN) T cells is characteristic for MRL/*lpr* mice. These cells seem to be unresponsive to antigen-receptor stimulation and have no T-helper activity [38, 39]. Determination of DN T cells identified as CD3^+^B220^+^CD4^–^CD8^–^ cells demonstrated a significant reduction of this cell population in mice treated with high-dose irinotecan. No effect was seen for low-dose irinotecan compared to saline-treated MRL/*lpr* mice (Fig. 3b).

B-cell numbers identified as CD3^–^B220^+^CD138^–^ cells remained unchanged in both irinotecan-treated groups (Fig. 3c). By contrast, the expression of CD69 on B cells and the numbers of plasmablasts (CD3^–^B220^+^CD138^int^) were significantly decreased in MRL/*lpr* mice treated with high-dose irinotecan. For plasma cells (CD3^–^B220^–^CD138^+^), no significant effect of either high-dose or low-dose irinotecan compared to saline was observed (Fig. 3c).

**Fig. 4:**
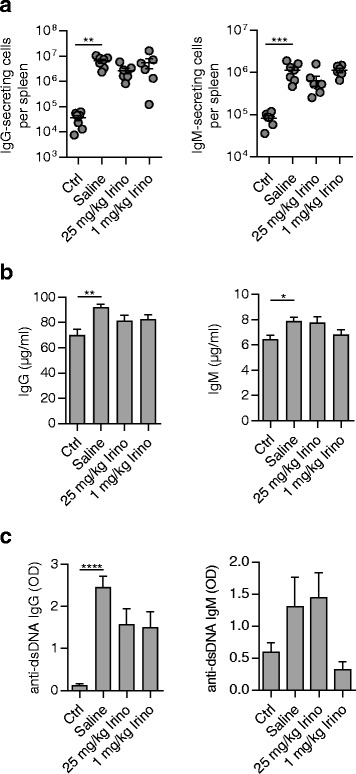
Decreased anti-double-stranded DNA (*dsDNA*) IgM in MRL/*lpr* mice treated with low-dose irinotecan (*Irino*). MRL/*lpr* mice receiving saline (*n* = 7), 25 mg/kg irinotecan (*n* = 6), or 1 mg/kg irinotecan (*n* = 6) were sacrificed at 18 weeks of age. MRL/MpJ mice treated with saline (*n* = 7) were used as controls (*Ctrl*). **a** Number of IgG- and IgM-secreting cells in the spleen determined by enzyme-linked immunosorbent spot assay. **b** Plasma levels of total IgG and IgM and **c** anti-dsDNA-specific IgG and IgM were measured by enzyme-linked immunosorbent assay. In (**a**), each symbol represents an individual mouse; bars show mean ± SEM. *P < 0.05, **P < 0.01, ***P < 0.001, versus saline-treated MRL/*lpr* mice by one-way analysis of variance with Bonferroni post hoc test

Next, we analyzed the numbers of antibody-secreting cells in the spleen. Surprisingly, increased numbers of IgG- and IgM-secreting cells in MRL/*lpr* mice were not affected by high- or low-dose irinotecan (Fig. 4a). In line with that, no changes in the plasma levels of total IgG and IgM were detected (Fig. 4b). Measurement of anti-dsDNA IgGs showed increased plasma levels in saline-treated MRL/*lpr* mice which were not significantly reduced in both irinotecan-treated groups (Fig. 4c, left). Unexpectedly, when analyzing anti-dsDNA IgM we found diminished, albeit not significantly, plasma levels in MRL/*lpr* mice treated with low-dose irinotecan while there was no reduction of anti-dsDNA IgM in MRL/*lpr* mice treated with high-dose irinotecan (Fig. 4c, right).

### High-dose irinotecan decreased the number of DN T cells infiltrating the lung

**Fig. 5:**
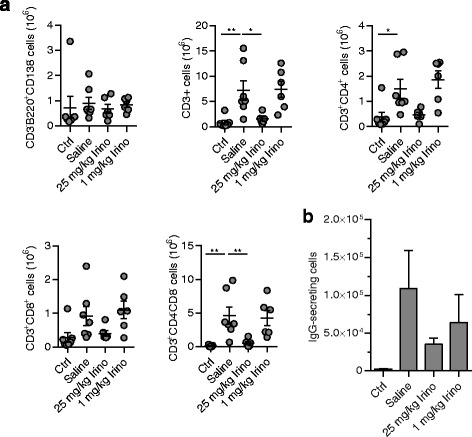
Reduction of infiltrating, autoreactive T cells in the lungs of MRL/*lpr* mice treated with high-dose irinotecan (*Irino*). Lungs were harvested from 18-week-old MRL/*lpr* mice that received saline (*n* = 7), 25 mg/kg irinotecan (*n* = 6), or 1 mg/kg irinotecan (*n* = 6). MRL/MpJ mice treated with saline (*n* = 7) were used as controls (*Ctrl*). **a** Lung cells were analyzed by flow cytometry for B cells (CD3^–^B220^+^CD138^–^) and T-cell subsets (CD4^+^, CD8^+^, and DN). **b** Total numbers of IgG-secreting cells isolated from the lungs were determined by enzyme-linked immunosorbent spot assay. In (**a**), symbols represent individual mice; bars show mean ± SEM.*P < 0.05, **P < 0.01, ***P < 0.001, versus saline-treated MRL/*lpr* mice by one-way analysis of variance corrected with Bonferroni’s multiple comparisons test

In addition to inflammatory processes in the kidneys and skin, MRL/*lpr* mice also exhibit pulmonary autoimmune disease which is caused by infiltration of mononuclear cells [40]. Analysis of different immune cell subsets in the lungs of 18-week-old MRL/*lpr* mice revealed a significant reduction of the number of CD3^+^ cells and DN T cells in the group treated with high-dose irinotecan (Fig. 5a). Moreover, there was some reduction in IgG-secreting lung cells in both irinotecan-treated groups; however, the difference did not reach statistical significance (Fig. 5b).

### Increased binding of anti-dsDNA antibodies to DNA modified by topo I was restricted to the IgG isotype

**Fig. 6:**
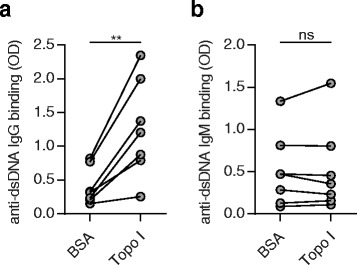
Increased binding of anti-double-stranded DNS (*dsDNA*) IgG but not of anti-dsDNA IgM to DNA treated with topoisomerase I (*topo I*). Calf thymus DNA was incubated with recombinant topo I or bovine serum albumin (*BSA*) for 30 min. Binding affinity of **a** anti-dsDNA IgG or **b** anti-dsDNA IgM from MRL/*lpr* plasma to modified DNA was assessed by enzyme-linked immunosorbent assay. Data were pooled from three independent experiments. **P < 0.01, by paired *t* test. *ns* not significant, *OD* optical density

Previous data from our group established a link between the extent of DNA relaxation and anti-dsDNA antibody binding. We demonstrated that anti-dsDNA antibody binding to DNA relaxed by recombinant topo I was clearly enhanced. This effect was shown for different types of DNA and for anti-dsDNA antibodies from different sources [21, 22]. To clarify whether this effect can be also applied to anti-dsDNA antibodies derived from MRL/*lpr* mice, we modified calf thymus DNA with recombinant topo I and assessed anti-dsDNA IgG binding by ELISA. Compared to BSA, DNA relaxed by topo I revealed a significantly increased binding of anti-dsDNA IgG from the plasma of MRL/*lpr* mice (Fig. 6a), in line with our previous observations. For the first time we also determined the binding of anti-dsDNA IgM to DNA modified by topo I. Applying the same conditions as described for anti-dsDNA IgG binding, we found that topo I-mediated DNA relaxation did not increase the binding of anti-dsDNA IgM derived from the plasma of MRL/*lpr* mice (Fig. 6b). Therefore, increased anti-dsDNA antibody binding following DNA relaxation seems to be restricted to anti-dsDNA IgG, which was an unexpected finding.

## Discussion

Five years ago, our group reported for the first time that the topo I inhibitor irinotecan is able to suppress lupus nephritis in NZB/NZW mice [20], a finding that was based on serendipity obtained from cancer research experiments. In those experiments, immunocompetent mice were treated with a human protein either alone or in combination with irinotecan. During the second cycle of treatment, all mice from the group treated with the protein alone died within 3 days whereas all mice treated with the protein in combination with irinotecan survived. In the review of these unexpected results we speculated about the occurrence of some form of fatal hypersensitivity reaction and a protective effect of irinotecan against this. Based on this hypothesis we introduced a mouse model for anaphylaxis in terms of fast hypersensitivity reaction. However, irinotecan failed to delay the onset of anaphylactic shock (unpublished results). We next checked whether protection from delayed hypersensitivity reactions was involved and established two mouse models of spontaneous autoimmune disease: diabetes in NOD mice and SLE in NZB/NZW. While irinotecan had no effect on the development of autoimmune diabetes (unpublished data), application of the topo I inhibitor almost completely prevented the onset of lupus nephritis. Encouraged by this finding we also examined whether irinotecan ameliorates the course of two other autoimmune diseases, multiple sclerosis and systemic sclerosis. Both attempts failed (unpublished results) providing evidence that inhibition of topoisomerase I is a specific treatment for SLE. This suggestion was supported by further experiments showing that extremely low concentrations of irinotecan still suppressed lupus nephritis and prolonged survival in NZB/NZW mice [21]. Final support for the potential use of irinotecan as a novel treatment strategy for SLE patients comes from our findings showing that irinotecan, even at a low dose, does not result in profound immunosuppression [20–22]. Instead, we presented evidence that alterations in DNA relaxation and subsequent changes in binding of anti-dsDNA antibodies may account for irinotecan-mediated effects in SLE [21, 22].

Are these data sufficient enough to legitimize first clinical trials treating lupus patients with irinotecan? Yes, we think so. However, to provide additional support in favor of clinical testing of irinotecan in human SLE, we introduced the MRL/*lpr* mouse model which is genetically different from NZB/NZW [41]. MRL/*lpr* mice were treated with either high-dose or low-dose irinotecan. As a result, high-dose irinotecan clearly suppressed the development of lupus-associated glomerulonephritis and prevented the onset of skin lesions. When applying low-dose irinotecan to MRL/*lpr* mice, the results were more difficult to interpret. Undoubtedly, until week 20, low-dose irinotecan almost completely inhibited the onset of proteinuria, a time point when most of the MRL/*lpr* mice-utilizing studies have finished [42–45]. Skin lesions were prevented by low-dose irinotecan at least until week 18, which was confirmed through histopathology assessed by a blinded pathologist. After week 18, an increasing number of mice from the group treated with low-dose irinotecan had to be sacrificed due to a skin score ≥4. The remaining mice of this group were suppressed for proteinuria, suggesting that low-dose irinotecan was more efficient at inhibiting lupus nephritis than skin lesions. However, it is very likely that intermediate dosages of irinotecan exist that are considerably lower than the doses used for chemotherapy, but which are still sufficient to suppress both lupus nephritis and skin lesions in MRL/*lpr* mice. This needs to be clarified in upcoming experiments.

Further investigation is also warranted for the finding that topo I-mediated DNA relaxation increased binding of anti-dsDNA IgG but not binding of anti-dsDNA IgM. Notably, anti-dsDNA IgM is known to have a protective function in SLE [46–49]. However, our previous results showing that DNA relaxation and anti-dsDNA IgG binding can be inhibited by topo I inhibitors explain how irinotecan executes its protective function in SLE. Without the use of a topo I inhibitor, circulating DNA might be relaxed allowing increased binding of anti-dsDNA IgG followed by inflammatory disease. In contrast, when applying a topo I inhibitor, circulating DNA might be less relaxed and, subsequently, binding of anti-dsDNA IgG will be reduced. Then, assuming that binding of anti-dsDNA IgM is independent of DNA relaxation, more anti-dsDNA IgM with its lupus-protective function would be able to bind to circulating DNA. However, such a mechanism is very speculative thus far and needs to be preferentially shown in vivo.

Summarizing our results, inhibition of topo I is highly proficient at suppressing lupus nephritis and other symptoms of lupus-like disease in two different mouse models of spontaneous SLE. Although the mechanism is not fully understood yet, it seems that topo I inhibition represents a specific and targeted therapy for SLE beyond immunosuppression. Therefore, first clinical trials applying low-dose irinotecan to human SLE patients are urgently required.

## Conclusions

The topo I inhibitor irinotecan suppressed both lupus nephritis and lupus-like skin lesions in MRL/*lpr* mice. Immunosuppression was not significantly involved in irinotecan-mediated suppression of lupus-like disease in MRL/*lpr* mice. As an alternative mechanism for irinotecan-mediated effects on lupus-like disease, our data propose altered binding of pathogenic anti-dsDNA IgG as a result of changes in DNA relaxation.

In view of the highly conserved nature of topo I, and considering the application of extremely low dosages of the topo I inhibitor as well as the lack of immunosuppression, inhibition of topo I might be developed as the first targeted therapy for human SLE.

## Abbreviations

ANOVA: Analysis of variance
BSA: Bovine serum albumin
DN: Double-negative
dsDNA: Double-stranded DNA
ELISA: Enzyme-linked immunosorbent assay
ELISpot: Enzyme-linked immunosorbent spot
ESRD: End-stage renal disease
FCS: Fetal calf serum
NZB/NZW: New Zealand Black/New Zealand White
PBS: Phosphate-buffered saline
SLE: Systemic lupus erythematosus
ssDNA: Single-stranded DNA
topo I: Topoisomerase I

## References

[1] Hochberg MC, Perlmutter DL, Medsger TA, Steen V, Weisman MH, White B, Wigley FM (1995). Prevalence of self-reported physician-diagnosed systemic lupus erythematosus in the USA. Lupus.

[2] Lionaki S, Skalioti C, Boletis JN (2014). Kidney transplantation in patients with systemic lupus erythematosus. World J Transplant.

[3] Cervera R, Khamashta MA, Font J, Sebastiani GD, Gil A, Lavilla P, Mejia JC, Aydintug AO, Chwalinska-Sadowska H, de Ramon E (2003). Morbidity and mortality in systemic lupus erythematosus during a 10-year period: a comparison of early and late manifestations in a cohort of 1,000 patients. Medicine (Baltimore).

[4] Mok CC, Kwok RC, Yip PS (2013). Effect of renal disease on the standardized mortality ratio and life expectancy of patients with systemic lupus erythematosus. Arthritis Rheum.

[5] Ward MM (2009). Changes in the incidence of endstage renal disease due to lupus nephritis in the United States, 1996–2004. J Rheumatol.

[6] Clark WF, Sontrop JM (2008). What have we learned about optimal induction therapy for lupus nephritis (III through V) from randomized, controlled trials?. Clin J Am Soc Nephrol.

[7] Hahn BH, McMahon MA, Wilkinson A, Wallace WD, Daikh DI, Fitzgerald JD, Karpouzas GA, Merrill JT, Wallace DJ, Yazdany J (2012). American College of Rheumatology guidelines for screening, treatment, and management of lupus nephritis. Arthritis Care Res.

[8] Contreras G, Pardo V, Leclercq B, Lenz O, Tozman E, O’Nan P, Roth D (2004). Sequential therapies for proliferative lupus nephritis. N Engl J Med.

[9] Casey TP (1973). Azathioprine administration to NZB X NZW hybrid mice with lupus nephritis: beneficial effect complicated by development of malignant lymphomas. N Z Med J.

[10] Bernatsky S, Ramsey-Goldman R, Labrecque J, Joseph L, Boivin JF, Petri M, Zoma A, Manzi S, Urowitz MB, Gladman D (2013). Cancer risk in systemic lupus: an updated international multi-centre cohort study. J Autoimmun..

[11] Rovin BH, Furie R, Latinis K, Looney RJ, Fervenza FC, Sanchez-Guerrero J, Maciuca R, Zhang D, Garg JP, Brunetta P (2012). Efficacy and safety of rituximab in patients with active proliferative lupus nephritis: the Lupus Nephritis Assessment with Rituximab study. Arthritis Rheum.

[12] Mysler EF, Spindler AJ, Guzman R, Bijl M, Jayne D, Furie RA, Houssiau FA, Drappa J, Close D, Maciuca R, et al. Efficacy and safety of ocrelizumab in active proliferative lupus nephritis: results from the randomized, double-blind phase III BELONG study. Arthritis Rheum. 2013;65(9):2368-79. doi:10.1002/art.38037. 10.1002/art.38037 23740801

[13] Isenberg D, Gordon C, Licu D, Copt S, Rossi CP, Wofsy D. Efficacy and safety of atacicept for prevention of flares in patients with moderate-to-severe systemic lupus erythematosus (SLE): 52-week data (APRIL-SLE randomised trial). Ann Rheum Dis. 2015;74(11):2006-15. doi:10.1136/annrheumdis-2013-205067. Epub 2014 Jun 20. 10.1136/annrheumdis-2013-205067 PMC4680140 24951103

[14] Furie R, Nicholls K, Cheng TT, Houssiau F, Burgos-Vargas R, Chen SL, Hillson JL, Meadows-Shropshire S, Kinaszczuk M, Merrill JT (2014). Efficacy and safety of abatacept in lupus nephritis: a twelve-month, randomized, double-blind study. Arthritis Rheumatol.

[15] ACCESS Trial Group (2014). Treatment of lupus nephritis with abatacept: the abatacept and cyclophosphamide combination efficacy and safety study. Arthritis Rheumatol..

[16] Navarra SV, Guzman RM, Gallacher AE, Hall S, Levy RA, Jimenez RE, Li EK, Thomas M, Kim HY, Leon MG (2011). Efficacy and safety of belimumab in patients with active systemic lupus erythematosus: a randomised, placebo-controlled, phase 3 trial. Lancet.

[17] Merrill JT, van Vollenhoven RF, Buyon JP, Furie RA, Stohl W, Morgan-Cox M, Dickson C, Anderson PW, Lee C, Berclaz PY, et al. Efficacy and safety of subcutaneous tabalumab, a monoclonal antibody to B-cell activating factor, in patients with systemic lupus erythematosus: results from ILLUMINATE-2, a 52-week, phase III, multicentre, randomised, double-blind, placebo-controlled study. Ann Rheum Dis. 2016;75(2):332-40. doi:10.1136/annrheumdis-2015-207654. Epub 2015 Aug 20. 10.1136/annrheumdis-2015-207654 26293163

[18] Furie R, Petri M, Zamani O, Cervera R, Wallace DJ, Tegzova D, Sanchez-Guerrero J, Schwarting A, Merrill JT, Chatham WW (2011). A phase III, randomized, placebo-controlled study of belimumab, a monoclonal antibody that inhibits B lymphocyte stimulator, in patients with systemic lupus erythematosus. Arthritis Rheum.

[19] Isenberg DA, Petri M, Kalunian K, Tanaka Y, Urowitz MB, Hoffman RW, Morgan-Cox M, Iikuni N, Silk M, Wallace DJ. Efficacy and safety of subcutaneous tabalumab in patients with systemic lupus erythematosus: results from ILLUMINATE-1, a 52-week, phase III, multicentre, randomised, double-blind, placebo-controlled study. Ann Rheum Dis. 2016;75(2):323-31. doi:10.1136/annrheumdis-2015-207653. Epub 2015 Sep 3. 10.1136/annrheumdis-2015-207653 26338095

[20] Frese-Schaper M, Zbaeren J, Gugger M, Monestier M, Frese S (2010). Reversal of established lupus nephritis and prolonged survival of New Zealand black x New Zealand white mice treated with the topoisomerase I inhibitor irinotecan. J Immunol.

[21] Frese-Schaper M, Keil A, Steiner SK, Gugger M, Korner M, Kocher GJ, Schiffer L, Anders HJ, Huynh-Do U, Schmid RA (2014). Low-dose irinotecan improves advanced lupus nephritis in mice potentially by changing DNA relaxation and anti-double-stranded DNA binding. Arthritis Rheumatol.

[22] Keil A, Frese-Schaper M, Steiner SK, Korner M, Schmid RA, Frese S (2015). The topoisomerase I inhibitor irinotecan and the tyrosyl-DNA phosphodiesterase 1 inhibitor furamidine synergistically suppress murine lupus nephritis. Arthritis Rheumatol.

[23] Koiwai O, Yasui Y, Sakai Y, Watanabe T, Ishii K, Yanagihara S, Andoh T (1993). Cloning of the mouse cDNA encoding DNA topoisomerase I and chromosomal location of the gene. Gene.

[24] Cheng C, Kussie P, Pavletich N, Shuman S (1998). Conservation of structure and mechanism between eukaryotic topoisomerase I and site-specific recombinases. Cell.

[25] Koster DA, Croquette V, Dekker C, Shuman S, Dekker NH (2005). Friction and torque govern the relaxation of DNA supercoils by eukaryotic topoisomerase IB. Nature.

[26] Hsiang YH, Hertzberg R, Hecht S, Liu LF (1985). Camptothecin induces protein-linked DNA breaks via mammalian DNA topoisomerase I. J Biol Chem.

[27] Hsiang YH, Lihou MG, Liu LF (1989). Arrest of replication forks by drug-stabilized topoisomerase I-DNA cleavable complexes as a mechanism of cell killing by camptothecin. Cancer Res.

[28] Wu J, Liu LF (1997). Processing of topoisomerase I cleavable complexes into DNA damage by transcription. Nucleic Acids Res.

[29] Pommier Y (2013). Drugging topoisomerases: lessons and challenges. ACS Chem Biol.

[30] Stewart L, Ireton GC, Champoux JJ (1999). A functional linker in human topoisomerase I is required for maximum sensitivity to camptothecin in a DNA relaxation assay. J Biol Chem.

[31] Lisby M, Olesen JR, Skouboe C, Krogh BO, Straub T, Boege F, Velmurugan S, Martensen PM, Andersen AH, Jayaram M (2001). Residues within the N-terminal domain of human topoisomerase I play a direct role in relaxation. J Biol Chem.

[32] Frese S, Diamond B (2011). Structural modification of DNA—a therapeutic option in SLE?. Nat Rev Rheumatol.

[33] Hahn BH (2001). Lessons in lupus: the mighty mouse. Lupus.

[34] Sun Y, Chen HM, Subudhi SK, Chen J, Koka R, Chen L, Fu YX (2002). Costimulatory molecule-targeted antibody therapy of a spontaneous autoimmune disease. Nat Med.

[35] Weening JJ, D’Agati VD, Schwartz MM, Seshan SV, Alpers CE, Appel GB (2004). on behalf of the International Society of Nephrology and Renal Pathology Society Working Group on the Classification of Lupus Nephritis. The classification of glomerulonephritis in systemic lupus erythematosus revisited. Kidney Int.

[36] Mizui M, Koga T, Lieberman LA, Beltran J, Yoshida N, Johnson MC, Tisch R, Tsokos GC (2014). IL-2 protects lupus-prone mice from multiple end-organ damage by limiting CD4–CD8- IL-17-producing T cells. J Immunol.

[37] Watanabe-Fukunaga R, Brannan CI, Copeland NG, Jenkins NA, Nagata S (1992). Lymphoproliferation disorder in mice explained by defects in Fas antigen that mediates apoptosis. Nature.

[38] Davignon JL, Budd RC, Ceredig R, Piguet PF, MacDonald HR, Cerottini JC, Vassalli P, Izui S (1985). Functional analysis of T cell subsets from mice bearing the lpr gene. J Immunol.

[39] Singer GG, Abbas AK (1994). The fas antigen is involved in peripheral but not thymic deletion of T lymphocytes in T cell receptor transgenic mice. Immunity.

[40] Kim N, Ussin L, Cheng X, Murali R, Sullivan KE (2002). TNFalpha inhibition in MRL/lpr mice ameliorates pulmonary but not renal disease. J Autoimmun.

[41] Kono DH, Theofilopoulos AN (2006). Genetics of SLE in mice. Springer Semin Immunopathol.

[42] Barber DF, Bartolome A, Hernandez C, Flores JM, Redondo C, Fernandez-Arias C, Camps M, Ruckle T, Schwarz MK, Rodriguez S (2005). PI3Kgamma inhibition blocks glomerulonephritis and extends lifespan in a mouse model of systemic lupus. Nat Med.

[43] Li Y, Chen F, Putt M, Koo YK, Madaio M, Cambier JC, Cohen PL, Eisenberg RA (2008). B cell depletion with anti-CD79 mAbs ameliorates autoimmune disease in MRL/lpr mice. J Immunol.

[44] Ahuja A, Shupe J, Dunn R, Kashgarian M, Kehry MR, Shlomchik MJ (2007). Depletion of B cells in murine lupus: efficacy and resistance. J Immunol.

[45] Rodgers DT, McGrath MA, Pineda MA, Al-Riyami L, Rzepecka J, Lumb F, Harnett W, Harnett MM (2015). The parasitic worm product ES-62 targets myeloid differentiation factor 88-dependent effector mechanisms to suppress antinuclear antibody production and proteinuria in MRL/lpr mice. Arthritis Rheumatol.

[46] Uher F, Puskas E, Cervenak J (2000). Beneficial effect of a human monoclonal IgM cryoglobulin on the autoimmune disease of New Zealand black mice. Cell Immunol.

[47] Werwitzke S, Trick D, Kamino K, Matthias T, Kniesch K, Schlegelberger B, Schmidt RE, Witte T (2005). Inhibition of lupus disease by anti-double-stranded DNA antibodies of the IgM isotype in the (NZB x NZW)F1 mouse. Arthritis Rheum.

[48] Stoehr AD, Schoen CT, Mertes MM, Eiglmeier S, Holecska V, Lorenz AK, Schommartz T, Schoen AL, Hess C, Winkler A (2011). TLR9 in peritoneal B-1b cells is essential for production of protective self-reactive IgM to control Th17 cells and severe autoimmunity. J Immunol.

[49] Johnson AC, Davison LM, Giltiay NV, Vareechon C, Li X, Jorgensen TN (2012). Lack of T cells in Act1-deficient mice results in elevated IgM-specific autoantibodies but reduced lupus-like disease. Eur J Immunol.

